# Lifetime extension and the recent cause of death in Werner syndrome: a retrospective study from 2011 to 2020

**DOI:** 10.1186/s13023-022-02383-w

**Published:** 2022-06-13

**Authors:** Hisaya Kato, Masaya Koshizaka, Hiyori Kaneko, Yoshiro Maezawa, Koutaro Yokote

**Affiliations:** 1 Department of Endocrinology, Hematology and Gerontology, Chiba University Graduate School of Medicine, 1-8-1 Inohana, Chuo-ku, Chiba, 260-8670 Japan; 2 Division of Diabetes, Metabolism and Endocrinology, Chiba University Hospital, 1-8-1 Inohana, Chuo-ku, Chiba, 260-8670 Japan

**Keywords:** Werner syndrome, Lifespan, Mortality, Epidemiology, Etiology, Rare disease, Ageing

## Abstract

**Background:**

Werner syndrome (WS) is an autosomal recessive premature ageing disease that causes accelerated ageing-like symptoms after puberty. Previous studies conducted in the late 2000s reported that malignant neoplasms and atherosclerotic diseases were the two leading causes of death, with life expectancies in the mid-50 s. However, the recent lifespan and cause of death in patients with WS remain unclear.

**Objective:**

To clarify the latest lifespan and causes of death in patients with WS.

**Method:**

We conducted a questionnaire-based survey in 2020 among the primary doctors of WS patients who were identified in previous nationwide surveys in Japan and clarified the following: the age of WS patients (age of death, if the patient had already died), sex, and cause of death. Patients who died in 2010 or earlier were excluded from the analysis.

**Results:**

A total of 123 living patients were identified at the time of the survey in 2020. Fourteen WS patients died between 2011 and 2020, with a mean age of 59.0 ± 8.9 years (mean ± SD). The most common cause of death was non-epithelial tumours, accounting for eight deaths, while no patient died of atherosclerotic diseases.

**Conclusions:**

Compared to previous studies, this study suggests that the lifespan of patients with WS has been extended. Although there were no deaths due to atherosclerotic diseases, non-epithelial tumours were still the leading cause of death. Further development of screening and treatment methods for these tumours is required.

## Introduction

Werner syndrome (WS), also known as adult progeria, is a rare autosomal recessive premature ageing syndrome displaying signs of ageing, such as grey hair, hair loss, cataracts, and diabetes, early after puberty [1]. Previous studies conducted in the late 2000s reported that the average life expectancy was in the mid-50s, and malignant tumours and cardiovascular diseases were the two leading causes of death [1–3]. As part of the Japanese Werner Consortium, we have been working on the development of diagnostic criteria, establishment of patient registries, and genetic diagnosis of suspected cases in Japan [4–6]. In the course of these efforts, we hypothesized that the recent life expectancy of individuals with WS had been extended because a number of patients recently died at a relatively advanced age compared to past cases [7]. Hence, this study aimed to clarify the latest lifespan and cause of death in WS.

## Materials and methods

We conducted a questionnaire-based survey in 2020 among the physicians who were identified as primary doctors of patients with WS in previous Japanese nationwide surveys and genetic tests [4–6, 8], to determine the following: the patient's age (at the time of death, if the patient had already died), sex, and cause of death. Those who died in 2010 or earlier were excluded from the analysis.

## Results

A total of 143 physicians were contacted for the survey, and 105 (73.4%) responded. At the time of the survey in 2020, 123 patients with WS were alive (61 males and 62 females) with a confirmed diagnosis according to the Japanese diagnostic criteria [4], and the mean age was 51.9 ± 9.6 years (mean ± SD) (Table 1). The number and age of patients who died between 2011 and 2020 were 14 (5 males and 9 females) and 59.0 ± 8.9 years (mean ± SD), respectively. The causes of death were eight malignant non-epithelial neoplasms (including five haematological malignancies) (Table 2), three infections, two malignant epithelial neoplasms, and one unidentified.

**Table 1:** Summary of the results

	Mean age ± SD	Number of patients
Patients with WS who were alive in 2020	51.9 ± 9.6	123 (male, 61)
Patients with WS who died between 2011 and 2020	59.0 ± 8.9	14 (male, 5)
**Causes of death**		
Non-epithelial malignancy		8
Infection		3
Epithelial malignancy		2
Unidentified		1

**Table 2:** Breakdown of the malignancies that caused deaths

Diagnosis	Number of patients
**Non-epithelial malignancy**	
MDS/AML	3
AML	1
Non-gastric MALT lymphoma	1
Fibrosarcoma	1
Osteosarcoma	1
Melanoma	1
**Epithelial malignancy**	
Hepatocellular carcinoma	1
Lung cancer	1

## Discussion/conclusion

This study suggests that the life expectancy of WS patients has been extended. Studies conducted in 2006 and 2008 reported that the median lifespan was 54.3 years [1], and the average lifespan was 55.0 years [3]. Also, the average age of patients who died between 1997 and 2006 was 51.8 years [2]. In the present study, the mean age of patients who died between 2011 and 2020 was 59.0 years, suggesting a 4.0 to 7.2-year increase in the lifespan of patients with WS compared to the previous studies. This increase is higher compared to the increase in life expectancy at birth in the general population in Japan from 2006 (males, 79.0 years; females, 85.8 years) or 2008 (males, 79.3 years; females, 86.1 years) to 2020 (males, 81.6 years; females, 87.7 years) [9–11]. These results imply that there are some factors that have specifically improved the life expectancy of WS in addition to the general factors, such as healthcare improvement, which have contributed to lifespan extension in the general Japanese population.

Regarding the cause of death, Goto et al. previously reported in a retrospective study of patients with WS from 1917 to 2008 in Japan that cardiovascular diseases were the second leading cause of death, accounting for 30% of the total deaths [3]. However, in the present study, there were no apparent deaths from atherosclerotic diseases such as myocardial infarction or stroke. Koshizaka et al. reported that only 2.5% of patients in the WS registry in 2020 had angina pectoris, myocardial infarction, or stroke [5]. They suggested that this decrease in prevalence compared to the WS nationwide study in 2009, which showed that 18.5% of patients with WS had angina pectoris, myocardial infarction, or stroke, might be due to the improved long-term control of metabolic risk factors such as dyslipidemia, hypertension, and diabetes with better treatment modalities that developed in recent decades. In addition, in a recent autopsy of a patient with WS who died at age 76, there were almost no atherosclerotic changes in the cerebral vessels and no stenosis in the coronary arteries [7, 12]. A number of other recent case reports have also shown that atherosclerotic changes were absent or slightly present [13, 14]. These findings suggest that atherosclerotic diseases are being controlled in WS. This result is partially consistent with the trends in leading causes of death in the general Japanese population which showed a decrease in age-adjusted death rates of heart diseases per 100,000 population from 2005 (males, 83.7; females, 45.3) to 2019 (males, 62.0; females, 31.3) [15]. However, heart disease was still the second leading cause of death in 2019, accounting for 15.0% of total deaths in Japan [16]. These facts indicate that atherosclerotic diseases in patients with WS are susceptible to general treatment and may not occur via a WS-specific mechanism.

On the other hand, the incidence of malignancies, mainly non-epithelial tumours, remained problematic in our study, accounting for 71.4% (10 of 14 cases) of total deaths (Table 1). Goto et al. reported that the incidence ratio of non-epithelial to epithelial neoplasms was 1.5:1 in WS, which is remarkably higher than the usual ratio of 1:10 [3]. Especially, haematological malignancies accounted for 17.6% of all neoplasms in WS cases from 1996 to 2008 [3]. In another study, Lauper et al. reported in a systematic review that 9.3% of WS tumours were haematological malignancies [17]. However, in the present study, 50% (5 of 10 cases) of the tumours that caused death were haematological malignancies (Table 2). These results indicate that managing haematological malignancies in patients with WS has become a substantial problem. In the general Japanese population, however, age-adjusted mortality rates of malignant neoplasms per 100,000 population decreased from 2005 (males, 197.7; females, 97.3) to 2019 (males, 149.5; females, 83.7) [15], while malignant neoplasms remained the leading cause of death in 2019, accounting for 27.3% of the total deaths [16]. Interestingly, while haematological tumour deaths have been declining as well (2005, 22.4 per 100,000; 2019, 19.5 per 100,000), their share of total tumour deaths have increased slightly (2005, 16.0%; 2019, 17.3%) [18]. However, this prevalence is far smaller than the percentage of haematological tumour deaths in WS; tumour development in WS, unlike atherosclerotic disease, might depend on a WS-specific mechanism [19].

In this study, we found an extension of the lifespan in patients with WS, which might be attributed to a decrease in the incidence of cardiovascular diseases. Although cardiovascular diseases are being controlled, malignant neoplasms account for the majority of deaths; therefore, effective screening and treatment strategies are required.

## Data Availability

The datasets used and analyzed during the current study are available from the corresponding author upon reasonable request.

## Abbreviations

WS: Werner syndrome
SD: Standard deviation
MDS: Myelodysplastic syndrome
AML: Acute myeloid leukaemia
MALT: Mucosa-associated lymphoid tissue
